# Association of Myometrial Invasion With Lymphovascular Space Invasion, Lymph Node Metastasis, Recurrence, and Overall Survival in Endometrial Cancer: A Meta-Analysis of 79 Studies With 68,870 Patients

**DOI:** 10.3389/fonc.2021.762329

**Published:** 2021-10-21

**Authors:** Jianzhang Wang, Ping Xu, Xueying Yang, Qin Yu, Xinxin Xu, Gen Zou, Xinmei Zhang

**Affiliations:** ^1^Department of Gynecology, Women’s Hospital, School of Medicine, Zhejiang University, Hangzhou, China; ^2^Department of Obstetrics and Gynecology, Beijing Shijitan Hospital, Capital Medical University, Beijing, China

**Keywords:** endometrial cancer, myometrial invasion, lymphovascular space invasion, lymph node metastasis, recurrence, overall survival, meta-analysis

## Abstract

**Background:**

Myometrial invasion has been demonstrated to correlate to clinicopathological characteristics and prognosis in endometrial cancer. However, not all the studies have the consistent results and no meta-analysis has investigated the association of myometrial invasion with lymphovascular space invasion (LVSI), lymph node metastasis (LNM), recurrence, and overall survival (OS). Therefore, a meta-analysis was performed to evaluate the relationship between myometrial invasion and clinicopathological characteristics or overall survival in endometrial cancer.

**Materials and Methods:**

A search of Pubmed, Embase, and Web of Science was carried out to collect relevant studies from their inception until June 30, 2021. The quality of each included study was evaluated using Newcastle–Ottawa scale (NOS) scale. Review Manager version 5.4 was employed to conduct the meta-analysis.

**Results:**

A total of 79 articles with 68,870 endometrial cancer patients were eligible including 9 articles for LVSI, 29 articles for LNM, 8 for recurrence, and 37 for OS in this meta-analysis. Myometrial invasion was associated with LVSI (RR 3.07; 95% CI 2.17–4.35; *p* < 0.00001), lymph node metastasis (LNM) (RR 4.45; 95% CI 3.29–6.01; *p* < 0.00001), and recurrence (RR 2.06; 95% CI 1.58–2.69; *p* < 0.00001). Deep myometrial invasion was also significantly related with poor OS *via* meta-synthesis of HRs in both univariate survival (HR 3.36, 95% CI 2.35–4.79, *p* < 0.00001) and multivariate survival (HR 2.00, 95% CI 1.59–2.53, *p* < 0.00001). Funnel plot suggested that there was no significant publication bias in this study.

**Conclusion:**

Deep myometrial invasion correlated to positive LVSI, positive LNM, cancer recurrence, and poor OS for endometrial cancer patients, indicating that myometrial invasion was a useful evaluation criterion to associate with clinical outcomes and prognosis of endometrial cancer since depth of myometrial invasion can be assessed before surgery. The large scale and comprehensive meta-analysis suggested that we should pay more attention to myometrial invasion in clinical practice, and its underlying mechanism also deserves further investigation.

## Introduction

Endometrial cancer is the most prevalent gynecological malignancy in developed countries (1) and the sixth most common cancer in women with continuously increasing incidence and associated mortality (2). Myometrial invasion, lymphovascular space invasion (LVSI), lymph node metastasis (LNM), and recurrence are the important molecular events and clinical behaviors for endometrial cancer. Among them, myometrial invasion is the quietly early action of cancer cells. In addition, three-dimensional ultrasound and magnetic resonance imaging are applied for preoperative assessment of the depth of myometrial invasion (3), and frozen sections are used for intraoperative estimation (4). It is meaningful to classify patients with initial stages as low-risk or high-risk patients for surgical planning when the above diagnostic methods are becoming more accurate with a better specificity and sensitivity. Therefore, it deserves more attention on myometrial invasion in endometrial cancer.

Myometrial invasion is defined as the invasion of endometrial cancer cells into myometrium. The depth of invasion is critical to the evaluation of surgical-pathological staging. According to the International Federation of Gynecology and Obstetrics (FIGO) staging system, stage IA includes those tumors with myometrial invasion of less than 50% or without myometrial invasion, and stage IB refers to more than 50% of invasion into myometrium. Although the underlying mechanism of myometrial invasion is still unclear, it is one of critical considerations for the surgery types and therapeutic methods. This is because accumulated evidences show that myometrial invasion is related to LVSI, LNM, recurrence, and OS of endometrial cancer in different reports. However, there are some different sounds, and not all the studies share the similar results. Specifically, there were more patients with positive LVSI in superficial myometrial invasion group when compared to deep myometrial invasion group (5); there was no statistically significant difference for positive LNM between superficial and deep myometrial invasion groups (6). In addition, there was no statistically significant difference for recurrence between superficial and deep myometrial invasion groups (7, 8). Therefore, a further study is valuable.

Currently, it is still a mystery whether the above non-uniform results change our previous conclusions and consensus. Until now, there is no aggregated estimate about the relationship between myometrial invasion and LVSI, LNM, recurrence, and OS. This meta-analysis is aiming to further elucidate whether myometrial invasion correlates to LVSI, LNM, recurrence, and OS based on the data available so far. The meta-analysis of multiple clinical studies will provide comprehensive descriptions about myometrial invasion not only from the past to the present but also from clinicopathological characteristics to prognostic value. We hope that the study also could provide us more certainty and confidence in mention and further investigation of myometrial invasion of endometrial cancer.

## Materials and Methods

### Literature Search Strategy

Literature was searched from Pubmed, Embase, and Web of Science from their inception until June 2021. The study published only in English was further considered. The main search terms were formulated as follows: “endometrial cancer”, “endometrial carcinoma”, “endometrial tumor”, “uterine carcinoma”, “uterine cancer”, “endometrial neoplasms”, “myometrial invasion”, “myometrial infiltration”, “clinicopathological factors”, “lymphovascular space invasion”, “lymph node metastasis”, “prognostic marker”, “prognosis”, “overall survival”, “recurrence”, and “relapse”.

### Inclusion and Exclusion Criteria

The study had to meet the following inclusion criteria: (1) the patients only had endometrial cancer; (2) enough data about clinicopathological factors (myometrial invasion, LVSI, LNM, or recurrence) and/or related information to extract hazard ratio (HR) and standard error (SE) of lnHR for OS; (3) article was published in English. The exclusion criteria included the following terms: (1) reviews, meta-analysis, animal experiments, and case reports; (2) republished articles; (3) incomplete and unpublished studies; (4) the study did not meet the design. Two reviewers independently reviewed the literatures according to the predefined strategy and criteria. The articles were screened with two researchers independently (JW and PX). The disagreements were further settled through discussion and resolved by a third investigator when necessary.

### Data Extraction and Quality Assessment

Two investigators (JW and PX) were assigned to assess the eligibility of all studies. Moreover, a third investigator (XZ) resolved the disagreements when necessary. The following information from each study was extracted: first author, publication year, the region of the study population, the number of participants, design type. For LVSI, LNM and recurrence, and the numbers in case and control groups were extracted respectively. For OS, HR estimate with 95% confidence interval (CI) for OS was extracted. The quality of included studies was assessed using Newcastle–Ottawa scale (NOS) scale, and the score of the quality ranged from 0 to 9.

### Data Analysis

Version 5.4 software of Review Manager was applied for this meta-analysis. Risk ratio (RR) with 95% CI was pooled to investigate the association between myometrial invasion and clinicopathological features (LVSI, LNM, and recurrence). HR with 95% CI was combined to study the effect of myometrial invasion on OS. The HR was extracted directly when the HR with 95% CI was reported. SE was calculated using the equation: SElnHR = (lnUpperCI − lnLowerCI)/3.92 (9). If the article did not provide direct HR while Kaplan–Meier survival curve was shown, Engauge Digitizer software was performed to acquire HR with SE (10). A random-effects model was conducted if significant heterogeneity (*p* ≤ 0.1, I^2^ > 50%) was shown. Publication bias was evaluated by the shape of funnel plot. Statistically significant difference was pointed out when a *p* value was less than 0.05.

## Results

### Study Search Results

The predefined search strategy identified 1,385 records. After screening of titles and abstracts, 1,058 records were excluded including 35 non-English papers, 18 duplicated records, 208 review/meta/letter/abstract, 19 animal studies, and 778 irrelated literature. Full text of 327 articles was assessed, and 248 records were excluded including 8 studies with the same included patients, 11 basic research, and 229 articles without adequate data. Finally, 79 studies of total 68,870 patients were eligible (5–8, 11–85), including 9 articles for LVSI, 29 articles for LNM, 8 for recurrence, and 37 for OS. The included studies and Newcastle-Ottawa scores are presented in **Table 1**.

**Table 1:** Baseline characteristics of included studies.

Author	Year	Study period	Country	N	Design	Outcomes	Quality*
Watanabe et al. (5)	2019	2010–2015	Japan	88	P	LVSI	8
Rychlik et al. (6)	2020	2000–2018	Spain	477	R	LNM, Univar-HR	6
Pradhan et al. (7)	2012	1998–2007	Norway	56	R	Rec	7
Van der Putten et al. (8)	2015	2005–2011	The Netherlands	81	R	Rec	6
Abbink et al. (11)	2018	1999–2009	The Netherlands	157	R	Univar-HR, Multi-HR	7
Abu-Zaid et al. (12)	2018	2009–2013	Saudi Arabia	148	R	Multi-HR	7
Akbayir et al. (13)	2012	2002–2010	Turkey	192	R	LNM	6
Akiyama-Abe et al. (14)	2013	1999–2009	Japan	221	R	Univar-HR, Multi-HR	8
Altunpulluk et al. (15)	2014	2006–2014	Turkey	121	R	LNM	7
Ambros et al. (16)	1991	1970–1988	Maryland	102	R	LVSI	7
Aoyama et al. (17)	2019	2007–2013	Japan	197	R	LNM	7
Ayhan et al. (18)	1994	1981–1991	Turkey	183	R	Rec	7
Bendifallah et al. (19)	2015	2001–2012	France	523	R	LNM	7
Bonatz et al. (20)	1999	1985–1990	Germany	164	R	Multi-HR	7
Capozzi et al. (21)	2020	2007–2007	Italy	614	R	LVSI	7
Cetinkaya et al. (22)	2014	1996–2010	Turkey	247	R	LNM	7
Chen et al. (23)	2001	1993–1998	Taiwan	53	R	Rec	7
Chen et al. (24)	2020	2009–2019	Taiwan	92	R	Univar-HR	6
Cheng et al. (25)	2019	2011–2012	China	113	R	Multi-HR	7
Cuylan et al. (26)	2018	2001–2016	Turkey	172	R	Multi-HR	7
Erkaya et al. (27)	2017	2007–2015	Turkey	500	R	Multi-HR	6
Ghezzi et al. (28)	2010	2000–2009	Italy	336	R	Univar-HR, Multi-HR	8
Günakan et al. (29)	2019	2007–2017	Turkey	762	R	LNM	6
Hasengaowa et al. (30)	2005	1997–2002	Japan	109	R	Multi-HR	7
Hiura et al. (31)	2010	1987–2002	Japan	284	R	Multi-HR	6
Ino et al. (32)	2006	1992–2001	Japan	80	R	Univar-HR	6
Jorge et al. (33)	2016	2010–2012	USA	25,907	R	LVSI	8
Kang et al. (34)	2014	2000–2006	South Korea	957	R	LNM	7
Koskas et al. (35)	2013	2002–2010	France	305	R	LVSI	6
Kwon et al. (36)	2009	1996–2000	Canada	314	R	LNM	7
Kyo et al. (37)	2006	1995–2002	Japan	70	R	Multi-HR	6
Larson et al. (38)	1996	1987–1995	USA	125	R	LNM	6
Lee et al. (39)	2009	2002–2008	South Korea	834	R	LNM	8
Lee et al. (40)	2016	2000–2013	South Korea	172	R	LNM	7
Li et al. (41)	2018	2010–2013	China	143	R	Multi-HR	7
Li et al. (42)	2019	2010–2018	China	874	R	LNM, Multi-HR	6
Li (2) et al. (43)	2019	2014–2019	China	388	R	LNM	7
Lin et al. (44)	2019	2006–2013	Taiwan	337	R	Univar-HR, Multi-HR	6
Lindah et al. (45)	1994	1980–1987	Sweden	251	R	Multi-HR	6
Machida et al. (46)	2018	2008–2015	USA	611	R	LVSI	6
Mahdi et al. (47)	2015	2005–2012	USA	140	R	LNM	6
Matsuo et al. (48)	2015	2000–2013	USA	703	R	LVSI	6
Mhawech-Fauceglia et al. (49)	2012	2000–2010	USA	279	R	Multi-HR	8
Miyamoto et al. (50)	2013	1996–2005	Japan	84	R	Univar-HR, Multi-HR	6
Nakamura et al. (51)	2011	2007–2011	Japan	106	P	Multi-HR	7
Neal et al. (52)	2016	2005–2012	USA	205	R	Univar-HR	7
Njølstad et al. (53)	2015	2001–2011	Norway	539	R	LNM	8
Nomura et al. (54)	2006	1975–2004	Japan	841	R	LNM	7
Ohno et al. (55)	2005	1995–2002	Japan	70	P	Multi-HR	8
Panggid et al. (56)	2010	1999–2007	Thailand	136	R	LVSI, Rec	7
Patel et al. (57)	2007	1989–2003	Canada	107	R	Univar-HR	7
Pifer et al. (58)	2020	2017–2019	USA	438	R	LVSI	8
Sahin et al. (59)	2019	2007–2016	Turkey	185	R	Rec	8
Sal et al. (60)	2016	2000–2008	Turkey	59	R	Multi-HR	6
Sarı et al. (61)	2018	2007–2016	Turkey	280	R	LNM	7
Schink et al. (62)	1991	1979–1988	USA	142	R	LNM	7
Scott et al. (63)	2017	2003–2009	Canada	849	R	Multi-HR	8
Shen et al. (64)	2020	2006–2013	China	263	R	Univar-HR, Multi-HR	7
Siesto et al. (65)	2020	2009–2015	Italy	363	R	Univar-HR, Multi-HR	6
Sigurdsson et al. (66)	1998	1964–1985	Iceland	203	R	Multi-HR	6
Solmaz et al. (67)	2015	1995–2012	Turkey	827	R	LNM	7
Stalberg et al. (68)	2019	2010–2017	Sweden	959	P	LNM, Multi-HR	8
Stiekema et al. (69)	2017	1994–2014	Netherlands	88	P	Univar-HR	8
Tanaka et al. (70)	2013	NR	Japan	354	R	Multi-HR	6
Tang et al. (71)	1998	1979–1996	Japan	310	R	LNM	6
Taşkın et al. (72)	2017	2011–2014	Turkey	279	R	LNM	7
Taskiran et al. (73)	2006	1982–2002	Turkey	461	R	LNM	8
Todo et al. (74)	2013	2000–2008	Korea	281	R	LNM	6
Tuomi et al. (75)	2017	2007–2013	Finland	929	R	Rec	7
Urabe et al. (76)	2014	1990–2010	Japan	366	R	Univar-HR, Multi-HR	6
Vargas et al. (77)	2014	1988–2010	USA	19329	R	LNM	8
Wakayama et al. (78)	2018	2006–2013	Japan	189	R	Multi-HR	6
Yabushita et al. (79)	2001	1986–1995	Japan	36	R	Rec	6
Yamada et al. (80)	2021	2014–2015	Japan	67	P	Univar-HR	7
Yokoyama et al. (81)	1997	1988–1996	Japan	60	R	LNM	6
Zanfagnin et al. (82)	2019	1999–2008	USA	85	R	LNM	7
Zhang et al. (83)	2012	1989–2006	China	621	R	LNM	7
Zhao et al. (84)	2015	2007–2008	China	188	R	Multi-HR	7
Zhao et al. (85)	2019	NR	China	89	R	Univar-HR, Multi-HR	6

R, Retrospectively study; P, prospectively study; LVSI, lymphovascular space invasion; LNM, lymph node metastasis; Univar-HR, HR in univariate analysis; Multi-HR, HR in multivariate analysis. ^*^The quality was assessed using Newcastle–Ottawa scale (NOS) scale.

### Myometrial Invasion Is Associated With LVSI in Endometrial Cancer

**Figure 1:**
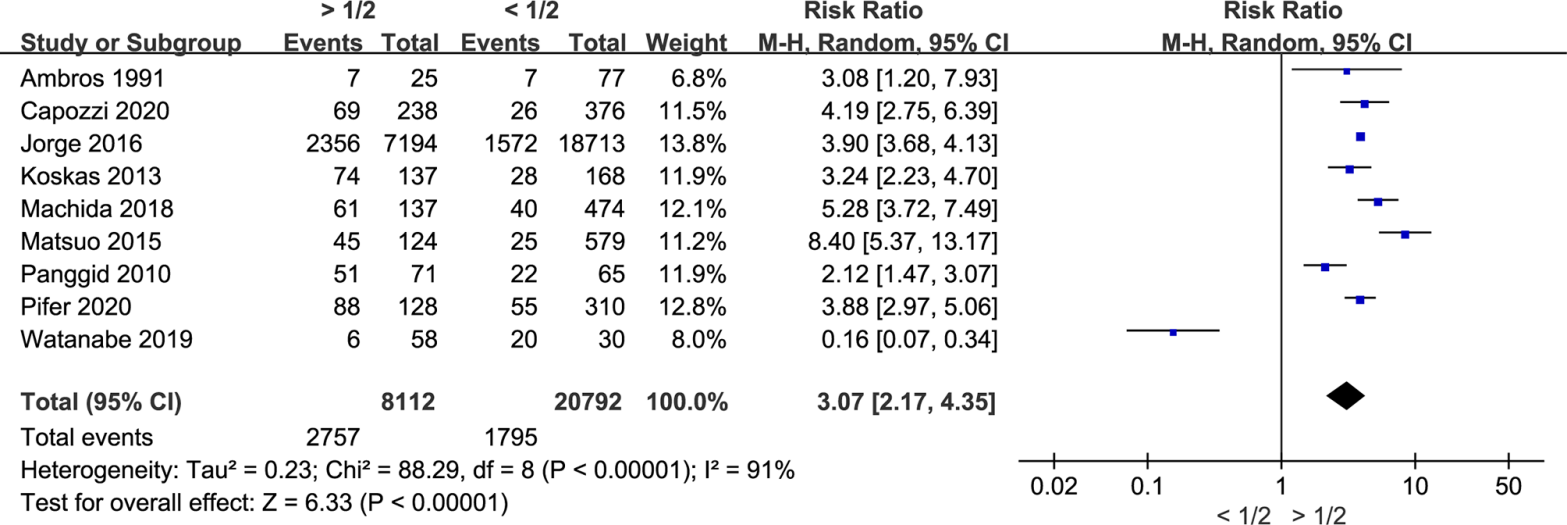
Meta-analysis of the association between myometrial invasion and LVSI in endometrial cancer.

Nine studies with a total of 28,904 endometrial cancer patients were finally included for this analysis. The random-effects model was applied due to the significant between-study heterogeneity (I^2^ = 91%, *p* < 0.00001). The risk ratio, which was expressed as >1/2 group *versus <*1/2 group, was 3.07 (CI 95% 2.17–4.35, *p* < 0.00001) (**Figure 1**). The pooled result showed that there was a link between the depth of myometrial invasion and the risk of LVSI. Combined with the clinical information from the included studies, the result indicated that patients with deeper myometrial invasion of endometrial cancer into myometrium (>1/2) were more prone to LVSI.

### Myometrial Invasion Is Associated With LNM in Endometrial Cancer

**Figure 2:**
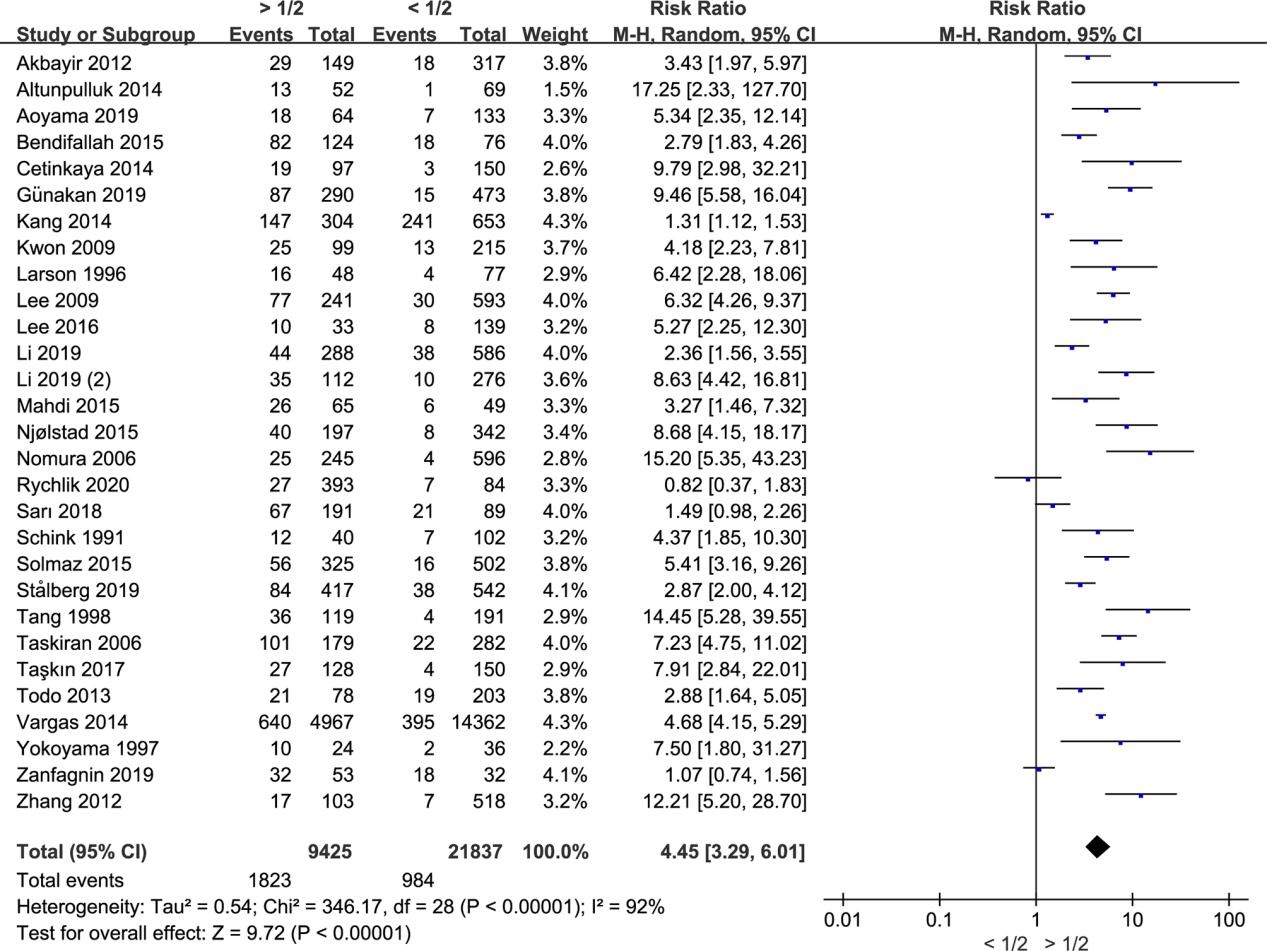
Meta-analysis of the association between myometrial invasion and LNM in endometrial cancer.

Twenty-nine studies including 31,262 endometrial cancer patients were eligible for analysis. The random-effects model was conducted for the significant between-study heterogeneity (I^2^ = 92%, *p* < 0.00001). The risk ratio was 4.45 (CI 95% 3.29–6.01, *p* < 0.00001) (**Figure 2**). The aggregated estimate of myometrial invasion was significantly associated with LVSI. According to the included studies, the result showed that deeper myometrial invasion is associated with the tendency of LNM in endometrial cancer.

### Myometrial Invasion Is Associated With the Recurrence of Endometrial Cancer

**Figure 3:**
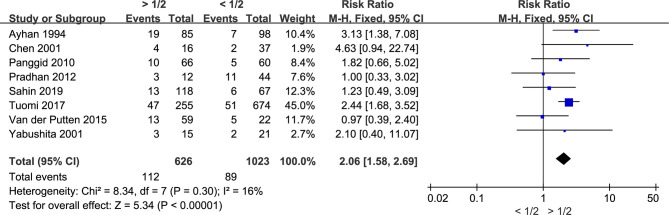
Meta-analysis of the association between myometrial invasion and recurrence in patients with endometrial cancer.

Since recurrence is the leading cause of death in cancers, further investigation is conducted by us on the association between myometrial invasion and the recurrence of endometrial cancer. Eight studies including 1,649 patients were included. During the analysis, we found that there was no significant between-study heterogeneity (I^2^ = 16%; *p* = 0.30), and fixed-effects model was used. Myometrial invasion was significantly associated with the recurrence of endometrial cancer since the risk ratio was 2.06 (CI 95% 1.58–2.69, *p* < 0.00001) (**Figure 3**). Therefore, deep myometrial invasion is associated with higher risk of endometrial cancer recurrence.

### Myometrial Invasion Is Associated With OS in Endometrial Cancer

**Figure 4:**
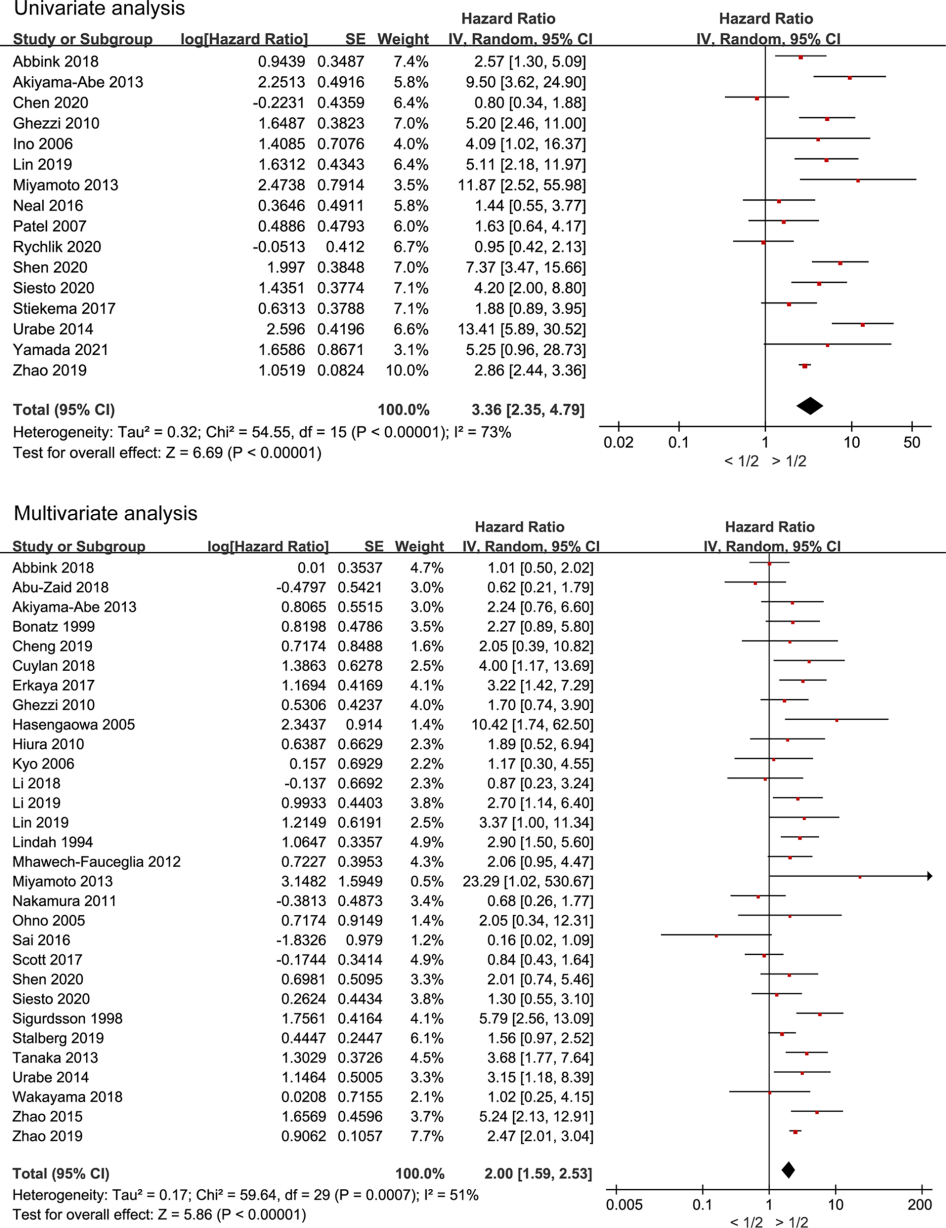
Meta-analysis of the association between myometrial invasion and overall survival in endometrial cancer patients according to HR from univariate or multivariate survival analyses.

Thirty-seven studies including 9,416 patients examined the association between myometrial invasion and OS in endometrial cancer. The pooled HRs of all-cause mortality with >1/2 myometrial invasion compared to <1/2 myometrial invasion were evaluated using random-effects model, and the results are presented in **Figure 4**. Pooled HRs of OS for univariate and multivariate analyses (HR 3.36, 95% CI 2.35–4.79, *P* < 0.00001, and HR 2.00, 95% CI 1.59–2.53, *P* < 0.00001, respectively) showed that the group with deep myometrial invasion was related with a higher risk of OS than the group with less than 1/2 myometrial invasion. Therefore, deep myometrial invasion is associated with poor survival in endometrial cancer.

### Publication Bias of Included Studies

**Figure 5:**
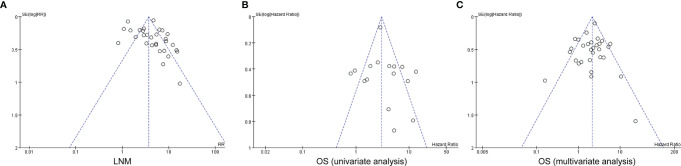
Funnel plots for potential publication bias. Funnel plot analysis of lymph node metastasis **(A)**, and univariate and multivariate survival analyses **(B, C)**. LNM, lymph node metastasis; OS, overall survival.

Funnel plot was applied for the assessment of publication bias in the literature, and tests for funnel plot asymmetry were applied only when there were at least 10 studies included in a meta-analysis. The shape of the funnel plot for the included 29 studies on the association between myometrial invasion and LNM was not significantly asymmetrical, indicating that there was no significant publication bias (**Figure 5A**). The results also show that no obvious publication bias was indicated in all included studies investigating myometrial invasion on OS in both univariate and multivariate analyses (**Figures 5B, C**).

## Discussion

Although there are many studies showing that myometrial invasion is definitely correlated to LVSI, LNM, recurrence, and OS, and we have reached an agreement that myometrial invasion is absolutely critical in the development of endometrial cancer, there are some inequable results, which makes us more or less feel lack confidence about that. Therefore, we searched all the studies about the relationship between myometrial invasion and clinicopathological characteristics (LVSI, LNM, and recurrence) or OS and conducted this meta-analysis.

The presence of LVSI is significantly associated with pelvic and paraaortic lymph node metastasis, recurrence, and poor prognosis (86, 87). As for lymph node metastasis, it is one of the evaluation criteria for the surgical-pathological staging and therapeutic schedule and is an extremely important determinant of the outcome. We paid extra attention to recurrence because it is uniformly associated with poor survival. Compared to LVSI, LNM, and recurrence, myometrial invasion is a much earlier molecular event and could be the initial driving force for the further progress of cancer cells. In addition, the depth of myometrial invasion before surgery can be accessed. Therefore, we should not only dig deeper into the underlying molecular mechanism but also pay more attention to the relevant clinical study. Since there are many studies about the relationship between myometrial invasion and clinicopathological characteristics (LVSI, LNM, and recurrence) or OS while not all the reports are consistent, we thereby pooled all the eligible studies and performed this meta-analysis.

Seventy-nine studies with a total of 68,870 endometrial cancer patients were finally included for this meta-analysis. Among them, nine studies with a total of 28,904 endometrial cancer patients were for LVSI. The pooled result showed patients with deeper myometrial invasion of endometrial cancer into myometrium (>1/2) were more prone to LVSI. As for LNM, 29 studies including 31,262 endometrial cancer patients were eligible for analysis, and the results demonstrated that deeper myometrial invasion is associated with the tendency of LNM in endometrial cancer. Furthermore, myometrial invasion was significantly associated with the recurrence of endometrial cancer according to the meta-analysis of eight studies including 1,649 patients. Since LVSI, LNM, and recurrence are independent prognostic factors for endometrial cancer patients, myometrial invasion would also be a prognostic factor. As it turned out, the group with deep myometrial invasion was related with a greater risk of OS than the group with less than 1/2 myometrial invasion based on not only univariate survival analysis but also multivariate survival analysis. Therefore, the results indicate that myometrial invasion is associated with LVSI, LNM, recurrence, and OS with much more confidence. Combined with preoperative assessment of the depth of myometrial invasion, now we know more information in regard to LVSI, LNM, recurrence, and OS of these patients before surgery, which suggests that we should especially pay more attention to myometrial invasion in clinical practice, and its underlying mechanism also deserves further investigation.

Potential limitations exist in this study, and meta-analysis without the classification of endometrial cancer is the obvious one. In the past, dualistic classification is the leading theory for the classification, which divides endometrial cancer into type I and type II tumors (88). According to histology, WHO classified endometrial cancer into the following subtypes: endometrioid, serous, mucinous, clear-cell, mixed, squamous-cell, transitional-cell, small-cell, and undifferentiated carcinomas (89). Among them, endometrioid carcinoma and serous carcinoma account for the majority. In this study, we check all the included 79 articles and found that histologic type was not only confined to endometrioid subtype although endometrioid carcinoma is the most common one. And 53 articles of the included 79 studies did not exclude other histologic types, so we did not further conduct the analysis based on histological classification. Recently, endometrial cancer is categorized into four genomic types: DNA polymerase epsilon (POLE) (ultramutated), microsatellite-instable (MSI) (hypermutated), copy-number low (endometrioid), and copy-number high (serous-like) tumors as the quick development of next-generation sequencing (90). The above genomic classification can facilitate the treatment tailored to specific subgroups and potentially enable the delivery of precision medicine to endometrial cancer patients. However, most included studies in the present meta-analysis did not perform the molecular classification, which may be because modern classification in molecular subtypes is relatively new and expensive, and thereby it is still not widely carried out in clinical practice. Given that, we appeal researchers to conduct more studies about the molecular classification and hope that more endometrial cancer patients benefit from the development of precision medicine.

Apart from classification, other potential limitations still exist in this study: (1) The data from the included studies were from the published articles instead of the original information of individual patient; (2) most included articles are the retrospective studies, and the evidence level is lower than that of prospective randomized clinical trial; (3) one of inclusion criteria is that article was published in English and negative results not being reported, which increase the risk of publication bias; (4) the number of included studies is relatively small, especially for LVSI and recurrence, which may cause biased results; (5) the heterogeneity of aggregated results was significant, and the random-effects model was applied.

## Conclusion

In summary, a large scale and comprehensive meta-analysis of the association between myometrial invasion and other clinicopathological characteristics and prognosis is provided in the present study. Our results show that myometrial invasion is associated with LVSI, LNM, recurrence, and OS, indicating that deep myometrial invasion is a useful evaluation criterion to associate with poor clinical outcomes and prognosis in endometrial cancer patients.

## Data Availability Statement

The original contributions presented in the study are included in the article/supplementary material. Further inquiries can be directed to the corresponding author.

## Author Contributions

JW, PX, and XZ: conceptualization. JW, PX, and XY: data curation and original draft writing. QY, XX, and GZ: statistical analysis. JW and XZ: manuscript review and editing. All authors contributed to the article and approved the submitted version.

## Funding

This study was supported by National Natural Science Foundation of China (Grant numbers: 81802591 and 81974225) and National Key R&D Program of China (Grant number: 2017YFC1001202).

## Conflict of Interest

The authors declare that the research was conducted in the absence of any commercial or financial relationships that could be construed as a potential conflict of interest.

## Publisher’s Note

All claims expressed in this article are solely those of the authors and do not necessarily represent those of their affiliated organizations, or those of the publisher, the editors and the reviewers. Any product that may be evaluated in this article, or claim that may be made by its manufacturer, is not guaranteed or endorsed by the publisher.
